# Peptide ion channel toxins from the bootlace worm, the longest animal on Earth

**DOI:** 10.1038/s41598-018-22305-w

**Published:** 2018-03-22

**Authors:** Erik Jacobsson, Håkan S. Andersson, Malin Strand, Steve Peigneur, Camilla Eriksson, Henrik Lodén, Mohammadreza Shariatgorji, Per E. Andrén, Eline K. M. Lebbe, K. Johan Rosengren, Jan Tytgat, Ulf Göransson

**Affiliations:** 1 Division of Pharmacognosy, Department of Medicinal Chemistry, Biomedical Center, Uppsala University, Box 574, SE-751 23 Uppsala, Sweden; 2 Linnaeus University Centre for Biomaterials Chemistry, Department of Chemistry and Biomedical Sciences, Linnaeus University, Kalmar, Sweden; 3 Swedish Species Information Centre, Swedish University of Agricultural Sciences, Uppsala, Sweden; 4 Toxicology & Pharmacology, University of Leuven (KU Leuven), O&N 2, PO Box 992, Herestraat 49, 3000 Leuven, Belgium; 5 Biomolecular Mass Spectrometry Imaging (BMSI), National and SciLifeLab Resource for Mass Spectrometry Imaging, Department of Pharmaceutical Biosciences, Biomedical Center, Uppsala University, Box 591, SE-751 24 Uppsala, Sweden; 6 School of Biomedical Sciences, The University of Queensland, Brisbane, QLD 4072 Australia

## Abstract

Polypeptides from animal venoms have found important uses as drugs, pharmacological tools, and within biotechnological and agricultural applications. We here report a novel family of cystine knot peptides from nemertean worms, with potent activity on voltage-gated sodium channels. These toxins, named the α-nemertides, were discovered in the epidermal mucus of *Lineus longissimus*, the ‘bootlace worm’ known as the longest animal on earth. The most abundant peptide, the 31-residue long α-1, was isolated, synthesized, and its 3D NMR structure determined. Transcriptome analysis including 17 species revealed eight α-nemertides, mainly distributed in the genus *Lineus*. α-1 caused paralysis and death in green crabs (*Carcinus maenas*) at 1 µg/kg (~300 pmol/kg). It showed profound effect on invertebrate voltage-gated sodium channels (*e.g. Blattella germanica* Na_v_1) at low nanomolar concentrations. Strong selectivity for insect over human sodium channels indicates that α-nemertides can be promising candidates for development of bioinsecticidal agents.

## Introduction

Peptides and proteins originating from animal venoms and toxins are intriguing sources of bioactive compounds. Some toxins have found their way to the market as drugs or pharmacological tools^1^, and others are finding applications in biotechnology and agriculture^2^. Snakes, scorpions, spiders, lizards, centipedes, and cone snails are all known producers of peptide toxins, but there are other classes of organisms for which the chemistry largely remains unknown. In the current work, we have explored one such neglected source of toxins: the nemerteans or ribbon worms.

Nemerteans have striking similarities with cone snails; both phyla use a proboscis for capture of prey, and one sub-class of nemerteans is also equipped with a stylet having the same apparent function as the radula tooth of the cone snail: venom injection. However, it is the mucus that covers the epidermis of nemerteans that appears to be the most conspicuous source of chemistry of these animals. Early indications of the bioactivity of the mucus have been attributed to the presence of low molecular weight toxins, including pyridyl alkaloids such as anabaseine^3^, and tetrodotoxin^4^.

In comparison, the interest in protein or peptide toxins from nemerteans has been sparse. Reports are confined to the cytolytic 10 kDa “A-toxins”, the 6 kDa neurotoxic “B-toxins” isolated from the mucus of the milky ribbon worm, *Cerebratulus lacteus*^5^, and the 10 kDa parborlysins that were discovered in *Parborlasia corrugatus*^6^. The molecular targets of these possible peptide toxins are yet unknown.

**Figure 1:**
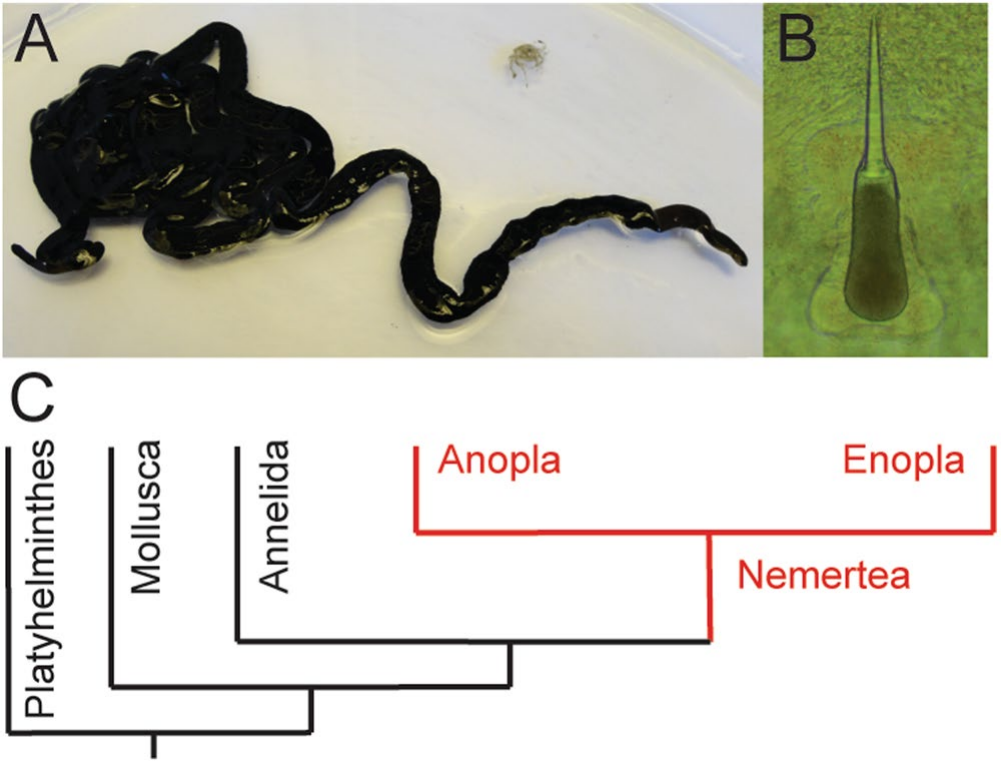
The nemertean *Lineus longissimus* and schematic of its phylogeny. (**A**) A specimen of *L. longissimus* (**B**) Example of a nemertean stylet apparatus (**C**) Stylized and simplified phylogenetic tree of *Lophotrochozoa*, displaying the relationship to *Mollusca*, wherein the cone snails are found, and the subdivision of the nemertea clade into enopla and anopla classes.

The *Nemertea* phylum comprises approximately 1300 species. *Lineus longissimus*, the bootlace worm, is known as the longest animal on earth with a body length of up to 50 m (Fig. 1). This species belongs to the anoplan nemerteans, carrying a proboscis without a stylet. This marine worm is found in the northern hemisphere at the intertidal sea bottom; on the Swedish west coast it is found from 10 m depths and below. *L. longissimus* first sparked our interest as a possible source of tetrodotoxin (TTX). However, TTX could never be identified, but the mucus still showed potent activity when injected into the branchial chamber of green crabs (*Carcinus maenas*)^7^. Instead, as shown in the current work, the activity is explained by the presence of a novel family of disulfide-rich peptide toxins.

## Results

### LC-MS analysis and MALDI-MSI show peptides in the mucus of *Lineus longissimus*

**Figure 2:**
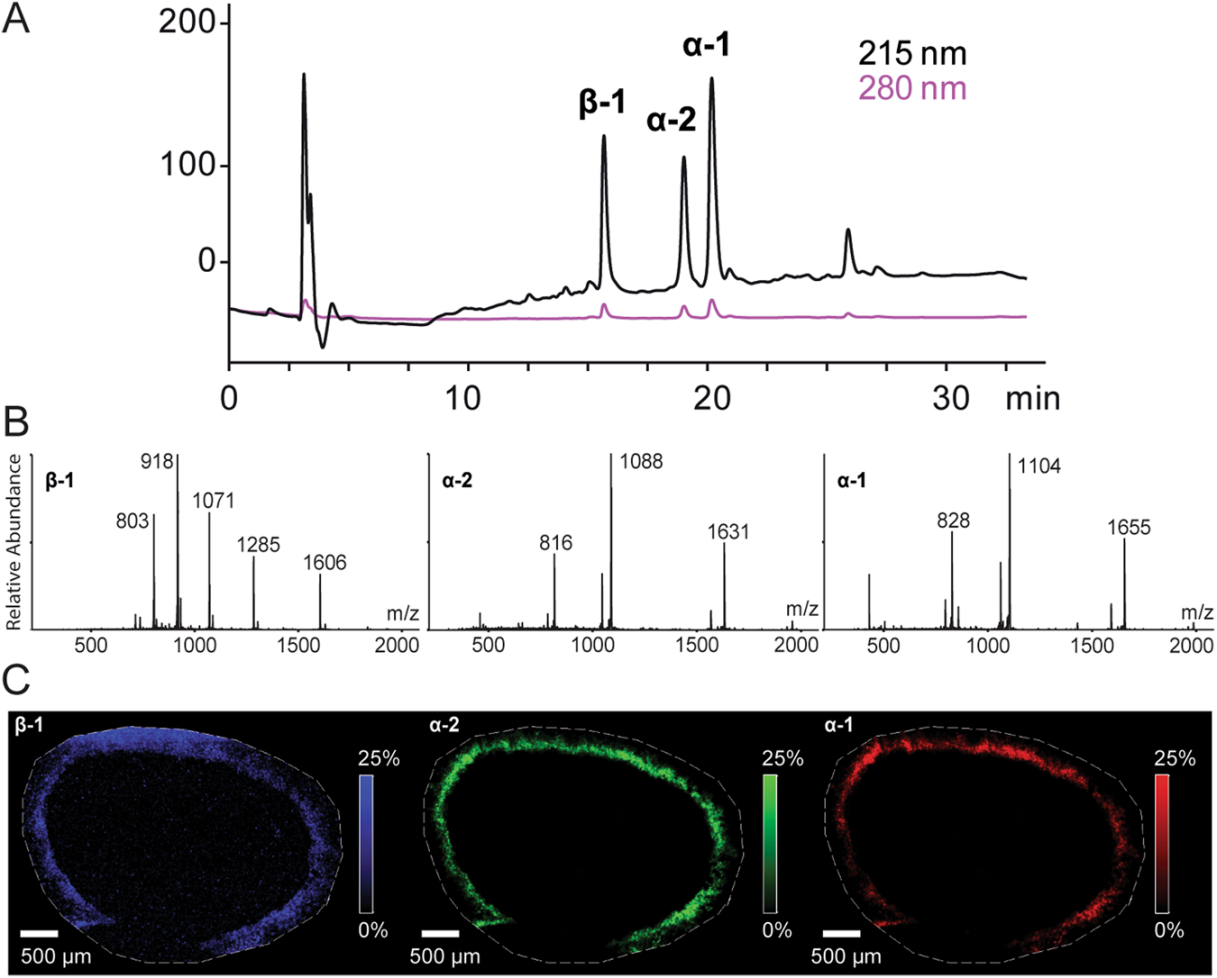
The discovery of nemertides. (**A**) RP-HPLC-UV trace of a high molecular weight fraction after SEC, showing nemertides α-1, α-2 and β-1. (**B**) MS spectra of the three peptides β-1 (left), α-2 (center) and α-1 (right). (**C**) The spatial distribution of nemertide β-1 (blue, 6419.821 ± 0.5 Da), α-2 (green, 3260.738 ± 0.5 Da) and α-1 (red, 3308.767 ± 0.5 Da) by MALDI-MSI (15 µm resolution) of a transversal section from the mid region of *L. longissimus*.

Specimens of *Lineus longissimus* were collected off the Swedish west coast, and kept alive in the laboratory. When provoked, this ribbon worm releases substantial amounts of mucus; this mucus was collected and lyophilized. After redissolving and desalting of the sample using gel filtration, HPLC and LC-MS analyses of the mucus (Fig. 2A) revealed three prominent peptide peaks with mass-to-charge (m/z) ratio (M + H^+^) of 3308.35, 3260.40 (mo.) and 6419.00 (av.) (Fig. 2B). These compounds were then isolated in µ-gram amounts using a combination of gel filtration and RP-HPLC. Matrix assisted laser desorption ionization mass spectrometry imaging (MALDI-MSI) analysis of cross sections demonstrated that the peptides are confined to the epidermis and to the mucus layer of the worm, Fig. 2C.

### Sequencing of nemertide peptides α-1 and α-2

**Figure 3:**
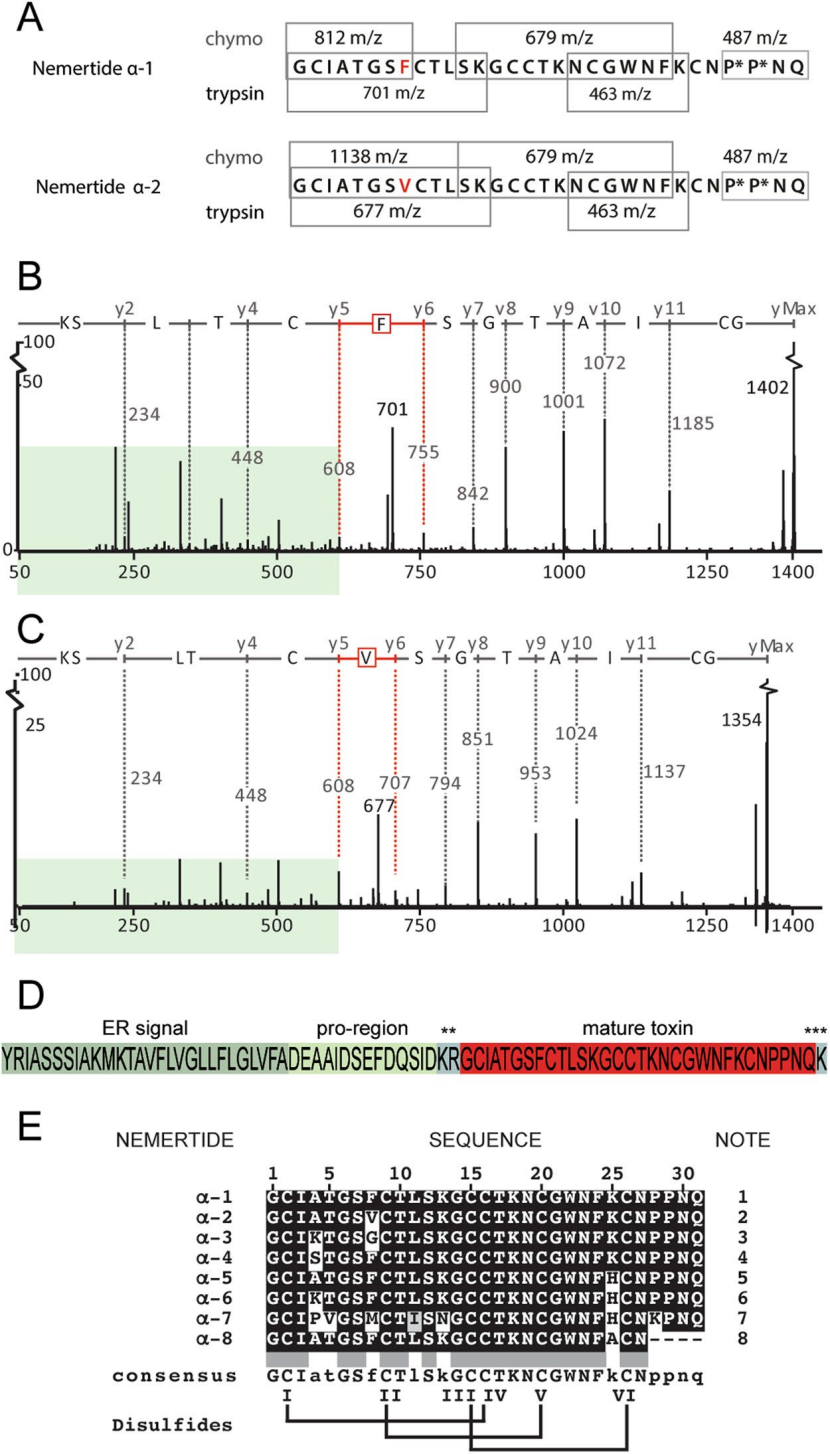
Sequencing and transcriptomic identification of α-nemertides. (**A**) Ion-map of enzymatically cleaved peptides sequenced by MSMS, *hydroxyproline. The C-terminal tryptic fragment (residues 26–31) of α-1 and α-2 did not give any sequence by MSMS. The 487 m/z fragment is consistent with P*P*NQ, which was found in both the native α-1 and -2 and confirmed by synthetic α-1. (**B**) and (**C**) Comparison of the N-terminal tryptic fragments of nemertides α-1 and α-2 demonstrate the change of F into V at position 8; the b and y ion series where peaks overlap are highlighted in pale green. Position 8 is highlighted in red in both spectra. All Cys residues were alkylated using iodoacetamide (Cys + 57). (**D**) α-1-precursor with the ER-signal, pro-region and mature toxin marked (predicted by Conoprec^38^. **Pre-sequence cleavage site, ***post-sequence lysine cleavage. (**E**) α-nemertides found in available transcriptomes. Disulfide connectivities in the sequences are inferred from the nemertide α-1. Notes: 1 present in *Lineus longissimus*, *L. lacteus* and *L. ruber*; 2 *L. longissimus*, *L. ruber*; 3 *L. lacteus*, *L. pseudolacteus*; 4 *L. sanguieneus*; 5 *L. pseudolacteus*; 6 *L. sanguieneus*; 7 *L. ruber*. One partial sequence was found in *R. occultus*, 8.

Isolated and purified peptides were reduced and alkylated, and the shifts in total mass demonstrated that the 3 kDa peptides contain three and the 6 kDa peptide four disulfide bonds. Quantitative amino acid analyses indicated that the 3 kDa peptides, named nemertide α-1 and α-2, are closely related. The 6 kDa peptide, which is distinct, was named nemertide β-1. For the α-family, experimental masses of both peptides differed from the masses calculated from amino acid analyses by 32 Da, suggesting further posttranslational modifications in addition to disulfide bond formation. Alkylated peptides were digested to generate fragments amenable for *de novo* sequencing using data from LC-MSMS, which resulted in the sequence for 20 out of the 31 amino acids to be successfully determined for α-1 and α-2 (Fig. 3).

In order to identity the missing segments, the transcriptome of *L. longissimus* was sequenced from RNA isolated from transversal dissections along the body of a single specimen. The assembled transcriptome contains 81597 contigs, with a total length of 91,851,747 bp. tBLASTn searches using the partial α-1 and α-2 sequences allowed identification of the full-length sequence of α-1. The full length of α-2 was subsequently identified using the transcriptome reported earlier by Whelan *et al*.^8^, confirming that the difference is confined to a single amino acid substitution of a Val to Phe. The combination of MSMS sequencing, amino acid analyses and transcriptome data suggested that the posttranslational modification involves hydroxylation of both Pro28 and Pro29, which explains the mass difference of 32 Da.

### Transcriptome analyses reveal α-, β-nemertides, A-toxins and parborlysins in nemerteans

The sequence of nemertide β-1 was also determined using transcriptome data. It comprises 57 amino acids, four disulfide bonds, and two hydroxyprolines (Hyp), and shows homology to neurotoxin B-II^9^. The transcriptomes of 17 species were subsequently analyzed for the presence of nemertides (Table S1). Seven complete α-nemertide sequences were discovered, all from the genus *Lineus* (Fig. 3), and one partial sequence from *Riseriellus occultus*. All α-nemertides discovered so far are from species within *Heteronemertea*, and no homologous sequences to α-nemertides were found in any other organisms.

The transcriptomes were also searched with queries for homologues to β-nemertides, A-toxins, parborlysins, and possible processing enzymes. Nemertide β-1 as identified in the *L. longissimus* transcriptome by matching MSMS data that was used as query together with the previously reported neurotoxin BII and BIV sequences. Eight additional β-nemertides (Figure S1A) were identified. Compared with α-nemertides, β-nemertides show a larger sequence variation, with an overall similarity of 51%. The A-toxin III (cytolysin A-III) has a sequence similarity with parborlysins 1–7 with a pairwise identity between 66.7 and 80.4%. In addition, 25 parborlysin homologues were found in addition to the seven sequences known previously (6) (Figure S1B). Interestingly, sequences resembling the proposed peptide maturation enzyme tex-31 from *Conus textile* were found in the *L. longissimus* transcriptome (Figure S2). Tex-31 cleaves a conotoxin propeptide with two basic residues in P1 and P2 positions, and preferably a leucine in P4 position^10^; its presence and likely processing site on the N-terminal side of mature α-1 nemertides suggests a possible common initial post-translational processing pathway.

### Synthesis and NMR structure of nemertide α-1

Nemertide α-1 was selected as a target for further characterization of the structural and functional features of this novel family of peptides. To obtain peptide in sufficient amounts for structural and biological characterization we turned to peptide chemical synthesis. Nemertide α-1 was assembled using FMOC solid phase peptide chemistry. The peptide was cleaved from the resin, yielding reduced linear α-1 as the main product (Figure S3A). Peptide could then be oxidatively folded, to a homogenous chromatographic compound. Co-elution studies using reverse phase HPLC demonstrated identical folds of synthetically produced and folded α-1 and the isolated native peptide (Figures S3B,C).

**Figure 4:**
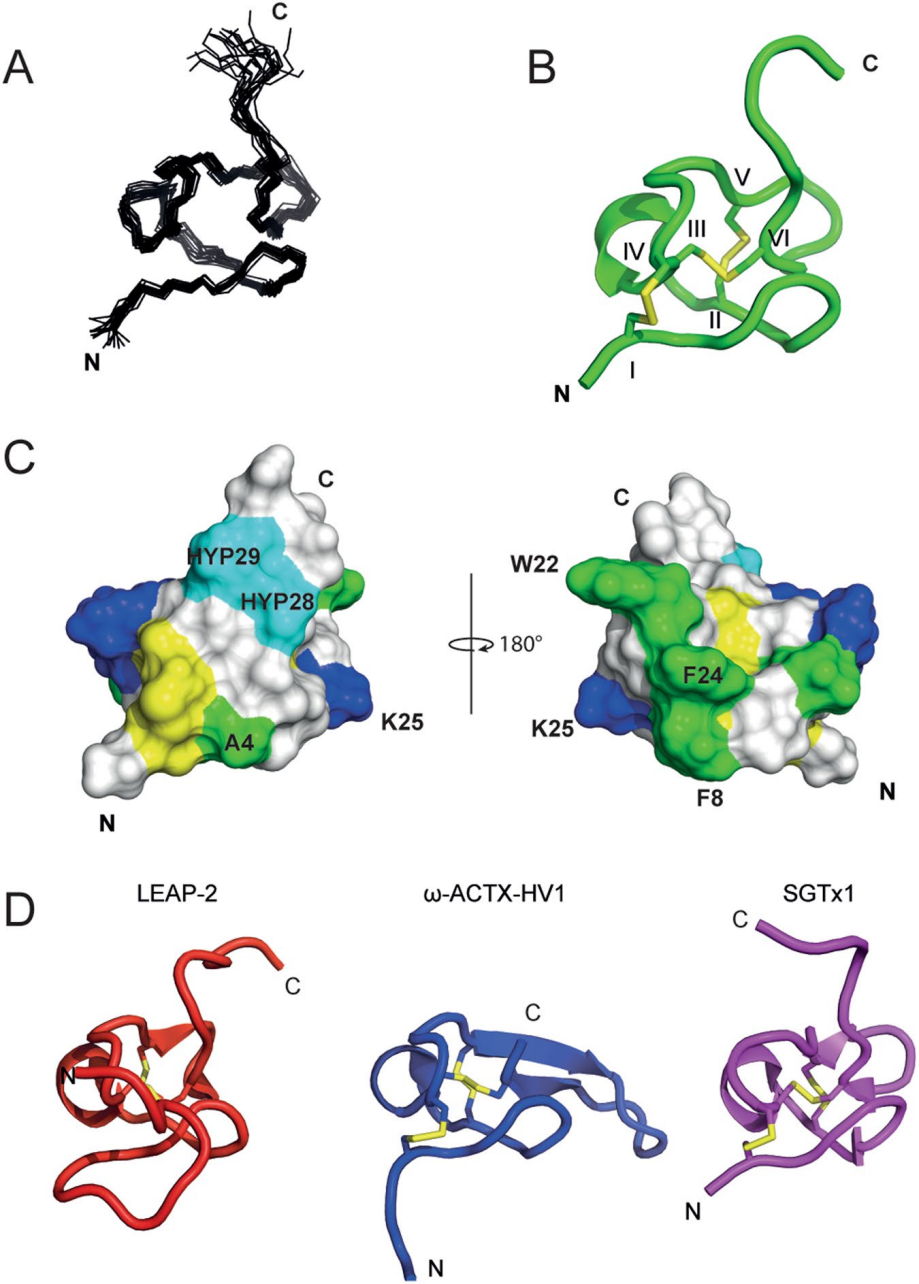
NMR structure of nemertide α-1 and identification of homologs. (**A**) Line representation of the 20 models with lowest MolProbity score. (**B**) Ribbon representation with disulfides (roman numbers), C and N-terminal displayed. (**C**) Surface model. Basic residues are shown in blue, nonpolar in green, cysteines in yellow and hydroxyprolines in cyan. The aromatic amino acids F8, F22, and W24 are labelled. F8 is the only difference between α-1 and -2. Structures are displayed using MOLMOL and PyMOL. PDB ID of nemertide alpha 1: 6ENA. **(D)** Closest structural homologs of α-1 identified from the PDB using the Daliserver: liver expressed antimicrobial peptide 2 (LEAP-2, PBD: 2l1q-A), ω-atracotoxin-HVIa (ω-ACTX-HV1, PBD: 1axh-A) and κ-theraphotoxin-Scg1a (SGTx1, PBD: 1la4-A).

The three-dimensional structure of α-1 was then determined using solution 2D NMR spectroscopy^11^ of the synthetic peptide. Overall, spectra were of excellent quality with well-dispersed signals indicating a defined structure (statistical data in Table S2). α-1 adapts a compact fold around the three disulfides located at the core of the molecule (Fig. 4). The disulfides form an inhibitor cystine knot (ICK) motif with a connectivity of Cys2-Cys16, Cys9-Cys20, and Cys15-Cys26. Sequence loops between cysteines are exposed at the surface of the molecule. The N-terminus is stabilized by the Cys2-Cys16 disulfide, whereas the C-terminus appears to be more flexible. The two Hyp residues are located in the C-terminal region. A short α-helix in the loop between Cys9 and Cys15 is the only element of regular secondary structure besides a series of tight turns. The two α-nemertides isolated from *L. longissimus* differ only by a Phe to Val substitution at position 8, which, together with Phe 22 and Trp 28, make up a surface exposed hydrophobic patch. In the family of α-nemertides, positions 4, 8 and 25 are subjects to variations. These residues are all displayed at the same side of the molecule. In analogy to other peptide toxins, these structural variations may control target preferences.

Structural alignment of α-1 using the DALI server^12^ identified human liver expressed antimicrobial peptide-2 LEAP-2 as the closest structural match with respect to Z score (Z: 3.7; rmsd: 1.1 Å; 21% sequence identity), followed by two ICK spider toxins, ω-HXTX-Hv1a (Z: 3.4; rmsd: 0.8; 30% sequence identity) and κ-TRTX-Scg1a (Z: 3.3; rmsd: 1.6; 20% sequence identity), Fig. 4D.

### Nemertide α-1 induces paralysis in green crabs and cockroaches

**Figure 5:**
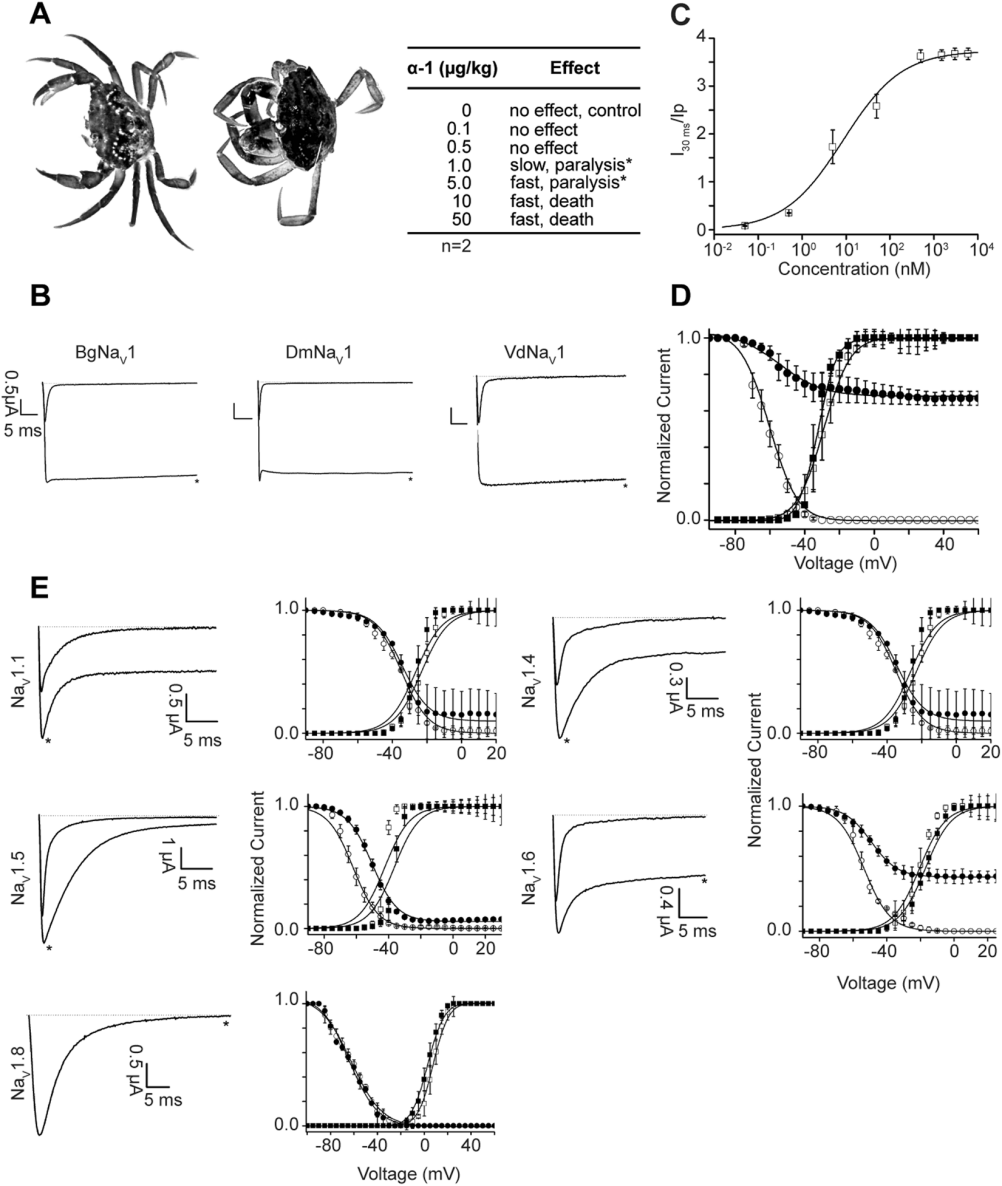
*In vivo* and *in vitro* activity of α-1. (**A**) α-1 injection in green crabs showing the hypertonic state; claws and legs pulled in towards the body, compared with control. Table shows dose response of α-1, all injections were made in duplicate (n = 2). *Animals did not recover. (**B**) Activity profile of α-1 on invertebrate Na_V_ channels of *Blattella germanica* (BgNa_V_1), *Drosophila melanogaster* (DmNa_V_1), *Varroa destructor* (VdNa_V_1). Dotted line indicates zero-current level; *marks steady-state current traces after application of 1 µM toxin (n ≥ 3). (**C**) Concentration-response curve for BgNa_V_1. EC_50_ is 8.6 ± 2.9 nM. (**D**) Steady-state activation and inactivation curves in control (open symbols) and toxin conditions (10 nM α-1, closed). No significant alteration of activation was noted. (**E**) Activity profile of α-1 on human Na_V_ channels. Left panels show whole-cell current traces in control and toxin conditions; dotted line indicates the zero-current level; *steady-state current traces after application of 6 µM α-1 (n ≥ 3). Right panels show steady-state activation (squares) and inactivation (circles) curves in control (open symbols) and toxin conditions (6 µM α-1, closed symbols). No significant alterations in the kinetics of gating were observed for Na_V_1.1 channels. Na_V_1.4: no shift in midpoint of activation; V_1/2_ inactivation shift −38.1 ± 0.2 mV to −48.6 ± 0.4 mV, in control and 6 µM α−1, respectively. Na_V_1.6: V_1/2_ activation shift −20,8 ± 0,1 mV to −17.3 ± 0.3 mV; V_1/2_ inactivation −53.6 ± 0.5 mV to −49.8 ± 0.2 mV. Na_V_1.5: V_1/2_ activation −42.4 ± 0,1 mV to −36.3 ± 0.1 mV; V_1/2_ inactivation −62.2 ± 0.2 mV in control and −50.6 ± 0.1 mV.

To deduce the biological function of α-nemertides we used an established model organism to study toxicity of nemertean mucus *in vivo*: the green crab *Carcinus maenas*^5,7^. This model has biological significance because crustaceans are both well-known preys and possible predators of nemerteans^13^. Injection of α-1 into green crabs resulted in tremor of the limbs within 1–2 minutes, followed by hypertonus and paralysis. After 20–30 minutes, hypertonus was released while paralysis remained. Paralysis occurred from 1 µg/kg while doses of 10 µg/kg led to death within minutes (Fig. 5). A complementary experiment was carried out on 50 specimens of juvenile *Blaptica dubia* cockroaches (Table S3). Injections of α-1 resulted in a similar response to that of the green crabs: after 24 hours, death or permanent paralysis was observed for all animals at doses higher than 7.1 µg/kg.

### Voltage-gated sodium channels are the targets of nemertide α-1

The rapid and potent paralytic effect of α-1 on *C. maenas*, the similarity of this effect to that of tetrodotoxin^14^, and the fact that ion channels are the most prominent targets of peptide toxins^2^, suggested that nemertide α-1 might assert its action by targeting voltage-gated sodium channels. Thus, synthetic α-1 was screened against three invertebrate and five vertebrate voltage-gated sodium channel isoforms (Na_V_s). Indeed, α-1 demonstrated a profound effect on the inactivation of invertebrate Na_V_ channels of *Blattella germanica* (BgNa_V_1), *Drosophila melanogaster* (DmNa_V_1) and *Varroa destructor* (VdNa_V_1) (Fig. 5). At 1 µM, α-1 completely inhibited their inactivation, resulting in sustained non-inactivating currents. The activity of α-1 was characterized in further details on BgNa_V_1 and its EC_50_ potency at the channel was found to be 8.6 ± 2.9 nM. Addition of 10 nM α-1 gave no significant alteration of activation, whereas for the inactivation curves, V_1/2_ shifted from −60.4 ± 0.6 mV in the control to −54.5 ± 1.6 mV in the presence of peptide. Furthermore, α-1 significantly enhanced the recovery from inactivation (Figure S4A), and pulsing experiments demonstrated that the open state is not required for the toxin to interact with the channel (Figure S4B). At the mammalian Na_V_s α-1 was found to be significantly less potent. Nonetheless, 6 µM nemertide α-1 delayed the inactivation of human Na_V_1.1, Na_V_1.4, Na_V_1.5 and Na_V_1.6, whereas no effect was seen on Na_V_1.8. The EC_50_ on Na_V_1.6 was determined to be 0.8 ± 0.1 µM (Figure S4C), highlighting that α-1 shows ~100-fold selectivity towards insect Na_V_s over tested mammalian Na_V_s. These data are encouraging for the further exploration of α-1 as a bioinsecticide.

## Discussion

In the current work we report the discovery of a new family of peptide toxins from nemertean worms. Eight homologous individual peptides were identified on peptide and/or transcriptome level. All but one peptide contain 31 amino acid residues, including three disulfide bonds arranged in an ICK motif. The sequences determined share less than 50% similarity with any yet described peptide. These so called α-nemertides were initially found at the peptide level in *Lineus longissimus*. The mucus of this species has strong effect in the green crab assay, which is explained by the high potency of the prototypic isolated peptide. The effect is striking, but to fully appreciate the potency it should be put into context and compared to peptides from other venomous animals against their target species. Among the arthropods, spiders are a rich source of peptide toxins targeting insects, and there is a growing interest in using these toxins for the development of bioinsecticides. The most potent of those toxins are active in the range of 10–100 pmol/g^2,^^15,16^. In comparison, the active dose of α-1 at 1 µg/kg is equivalent to 300 fmol/g, highlighting the extraordinary potency of α-1. The fact that similar effect at a similar range (7.1–14.7 µg/kg) was observed upon injection in *Blaptica* *dubia* cockroaches, suggests that α-1 might be a suitable framework for development of novel bioinsectides. Indeed, ICK peptides have recently been introduced for that purpose on the agricultural market^17^.

The prototypic nemertide α-1 was shown to exert its neurotoxic activity by slowing down the inactivation of Na_V_ channels, inducing a change of the steady-state inactivation without significant changes in the activation process. Upon binding, α-1 delays the recovery from inactivation. Furthermore, α-1 interacts with the Na_V_ channels in the closed state. The α-1 induced alterations of gating kinetics resemble the effects of other peptide toxins known to be binding to the so-called neurotoxin binding site 3^18,19^. Such toxins trap the voltage-sensing segment S4 of domain 4 in its inward or deactivated position, preventing fast inactivation^20,21^. Several toxins capable of binding to site 3 have been isolated from marine organisms^22^. Among these are the sea anemone toxins ATX-II and anthopleurin A that similarly act on Na_V_ channels, and like α-1 exert anti-insect specificity as demonstrated by the complete inhibition of the inactivation of the insect Na_V_ channels DmNa_V_1 and BgNa_V_1. However, additional experiments are needed to determine if site 3 is the target of α-1, for example competition experiments with known binders to that site.

Although the sequences of α-nemertides are unique, the ICK motif is shared with many other peptides, including toxins from spiders and cone snails and antimicrobial peptides. However, the closest 3D structural match is not an ICK peptide but LEAP-2. Backbone similarity of LEAP-2 and α-1 is striking, but LEAP-2 contains two disulfides only and patterns of surface hydrophobicity do not overlap. The physiological function of LEAP-2 is not clear^23^. Next structural matches are both spider toxins targeting voltage-gated calcium channels (ω-HXTX-Hv1a)^24^ and voltage-activated potassium channels (κ-TRTX-Scg1a)^25^. ω-HXTX-Hv1a belongs to the ω-atracotoxins, a family of arthropod-selective spider toxins initially characterized as selective insect Ca_V_ channel blockers. ω-HXTX-Hv1a has never been investigated for its activity on Na_V_ channels. However, it should be noted that ω-HXTX-Ar1a, which shares 84% identity with ω-HXTX-Hv1a, does block insect Na_V_ channels at higher concentrations (1 µM, 18% block)^26^. Together, this highlights how conserved folds and frameworks can adapt to different targets.

In comparison to other peptide toxins, the size of the peptides, the conserved ICK motif, and the origin and physiology of the expressing organism all draw clear parallels to cone snail toxins. In addition, sequences similar to the proposed peptide maturation enzyme tex-31 from *Conus textile*^10^ could be identified in the *L. longissimus* transcriptome (Figure S2). However, there are also profound differences: α-nemertides, despite their conserved structure, show little sequence similarity to any other peptide or protein, and whereas cone snail venoms contain complex libraries of hundreds to thousands of different peptides^27^, only two α-nemertides could be identified on peptide and RNA level in *L. longissimus*. In all, only eight different α-nemertide sequences could be identified from 17 transcriptomes, with no species expressing more than three variants. This is in stark contrast to the cocktails of toxins expressed by many other venomous animals, suggesting differences in the evolutionary pressure that have driven the expansion of the toxin repertoire in for example cone snails in which specialized sets of toxins are even used for capture of prey and for defense^28^. To us, the occurrence of the low number of toxins in e.g. *Lineus longissimus* suggests that they have a highly specialized function for the worm.

Whether α-nemertides are used for defense or capture of prey remains in question. The potent effect in the green crab assay does not exclude either explanation^13,29^ as crustaceans might be both prey and predator of nemerteans. In is noteworthy that selectivity differs between the toxins of cone snails and ribbon worms: whereas many cone snail peptides potently target vertebrate ion channels—and indeed are extensively pursued as clinically useful therapeutics—nemertide α-1 preferentially targets invertebrates. The further exploration of the *Nemertea* phylum may reveal further diversity in structures and activities among these toxins. To date, only a small fraction of the phylum has been investigated (the 17 species represent 1.5% of the known nemertean species).

The transcriptome analyses also revealed the presence of other peptide toxins, including novel parborlysin homologues. These are the first peptides of that family not originating from *Parborlasia corrugatus*, which has given these peptides their names. In the current work, we suggest the term “nemertides” as a general name for *nemer*tean pep*tides*, given the limited knowledge of their function, distribution and diversity. Moreover, because the physiological roles of neither the 3 kDa nor the 6 kDa nemertides reported here have yet been fully elucidated, we named peptide families α and β based on sequence similarities followed by a number indicating the order of discovery).

Bioinformatics was critical for the discovery of nemertides, but the key was the combination with biochemical analyses. This includes the extraction of peptides and analyses using chromatography and mass spectrometry. The prototypic α-1 peptide of this novel family was characterized with regard to structure and function, revealing striking activity on invertebrate voltage-gated sodium channels. In addition, the presence of these peptide toxins explains the bioactivity of the mucus previously attributed to TTX.

The results provide insight into the biological function of this new family of ICK peptides, and into the unknowns of chemical ecology of marine invertebrates. Indeed, nemertide α-1 is one of the most potent insecticidal peptides yet discovered, as judged by the activity on cockroach Na_v_s, indicating a potential use of α-nemertides in the development of novel insecticides.

## Materials and Methods

### Collection of *Lineus longissimus*

Living specimens were collected at the west coast of Sweden (Kosterfjord, 35 m depth) and identified by Dr Malin Strand. Specimens were placed in a beaker containing seawater, and released mucus was collected after gently agitating the animal with a glass rod. One specimen was cut into pieces, which were either flash-frozen in liquid nitrogen or placed in RNA-later® solution.

### Peptide Isolation

Lyophilized mucus was dissolved in 30% acetonitrile (AcN) in water and 0.1% formic acid (FA), and desalted using size exclusion chromatography (SEC; PD-10, GE Healthcare). The eluate was lyophilized and redissolved in 10% AcN, 0.1% FA, and subjected to RP-HPLC on a Phenomenex Jupiter column (5 µ C18 300 Å, 250 × 4.6 mm) using a Shimadzu LC20 system. The gradient ranged from 5 to 55% AcN, in 0.05% trifluoroacetic acid (TFA).

### LC-MS/MSMS

Dry, reduced and alkylated peptide was cleaved using trypsin, chymotrypsin or endoproteinase Glu-C, in separate experiments, Fig. 3. Peptides were analyzed using a UPLC-QToF nanospray MS (Waters nanoAcquity, QToF Micro; 75 µm × 250 mm 1.7 µm BEH130 C18). Scan window was set to 200–2500 m/z and for MSMS to 50–2000 m/z.

### Total RNA analysis

Total RNA was extracted from both flash-frozen and samples stored in RNAlater®, using Qiagen AllPrep DNA/RNA Mini Kit. The combined total RNA was sequenced using Illumina HiSeq. 2000 based RNA-seq paired end analysis and assembled by using Trinity (v 2011-11-26)^30^ (Macrogen, Korea).

### MALDI-MSI

Flash frozen tissues were cut (14 μm) using a cryostat-microtome (Leica CM3050S; Leica Microsystems, Welzlar, Germany), and thaw-mounted onto conductive indium tin oxide glass slides (Bruker Daltonics, Bremen, Germany). Sections were dried under a flow of nitrogen and desiccated at room temperature for 15 min, coated with 2,5-dihydroxybenzoic acid (35 mg/ml in 50% AcN, 0.2% TFA) before analysis using MALDI-TOF/TOF (Ultraflextreme, Bruker Daltonics). The Smartbeam II 2 kHz laser was operated in positive ion mode. Purified α-1, α-2 and β-1 were spotted on one section as an *in-situ* reference.

### Peptide synthesis

Nemertide α-1 was assembled on TentaGel XV HMPA resin (0.05 mmol scale) using Fmoc-based solid-phase peptide synthesis (SPPS). The C-terminal residues HypHypAsnGln and the dipeptide Fmoc-Leu-Ser(ψ^Me,Me^Pro)-OH in position 11–12 were coupled manually, the latter to prevent peptide chain aggregation. Remaining residues were assembled using automated microwave-assisted SPPS. Cleavage was performed using TFA/TIPS/H_2_O (95:2.5:2.5 v/v) for 2 hours. Crude peptide, Figure S3A, was subjected to oxidative folding in GSH:GSSG 2 mM: 0.4 mM in 0.1 M NH_4_HCO_3_ (pH 8.5), containing 20% (v/v) isopropanol, Figure S3B. The peptide was purified using a Phenomenex Jupiter C18 column (250 × 10 mm, 5 µ) and a gradient from 5 to 97% AcN, in 0.05% TFA. The pure synthetic peptide was subsequently co-injected with the isolated peptide in the HPLC system, to confirm correct folding, Figure S3C.

### NMR structure determination

Synthetic α-1 was dissolved in 90% H_2_O/10% D_2_O, and data were collected on a Bruker Avance 600 MHz spectrometer equipped with a cryoprobe. 2,2-Dimethyl-2-silapentane-5-sulphonate was used as internal standard (0.0 ppm). TOCSY, NOESY, ^13^CHSQC and ^15^NHSQC data were recorded at 298 K. TOCSY spectra were collected at 288–308 K with 5 K increments to establish temperature coefficients used for prediction of hydrogen bonds^31^. NMR spectra were assigned in CARA^32^. CYANA 3.0^33^ was used to assign NOE data and translate NOE intensities into distance restraints, with the final structures being refined in explicit water using CNS^34^. The 50 structures with lowest overall energies were evaluated with MolProbity^35^, and the 20 models with best stereochemistry chosen to represent the solution structure of α-1. PDB ID: 6ENA. Atomic RMSD was calculated over the residues between the first and last cysteine residues using MOLMOL^36^, Table S2. The Dali server^12^ was used to identify structural homologues, regardless of sequence similarity, Fig. 4D.

### *Carcinus maenas* Assay

Green crabs (20–50 g) were injected with control (500 µl sterile filtered sea water) or nemertide α-1 dissolved in sterile filtered sea water, into the cephalothorax between the first and second walking leg on the right side of the crab. Doses ranged from 0.1–50 µg/kg, in a maximal volume of 500 µl. The crabs were placed into a container filled with seawater and observed. All injections were made in duplicate.

### Heterologous Expression in *Xenopus laevis* Oocytes

Complementary DNA encoding the Na_V_-channels was subcloned into the corresponding vector: the α-subunits rNa_V_1.1/pLCT1(NotI), rNa_V_1.4/pUI-2(NotI), hNa_V_1.5/pcDNA3.1(XbaI), mNa_V_1.6/pLCT(NotI), cockroach *Blattella germanica* BgNa_V_1.1/pGH19(NotI), *Drosophila melanogaster* DmNa_V_1, *Varroa destructor* VdNa_V_1, and the corresponding β-subunits rβ1/pSP64T(EcoRI) and *Drosophila melanogaster* TipE/pGH19(NotI). The linearized plasmids—respective restriction enzymes are indicated in parentheses—were transcribed using the T7 (for rNa_V_1.1, rNa_V_1.4, mNa_V_1.6, BgNa_V_1.1, TipE) or the SP6 (for hNa_V_1.5 and rβ1) mMESSAGE-mMACHINE transcription kit (Ambion, Austin, TX). The harvesting of stage V-VI oocytes from anaesthetized female *Xenopus laevis* frogs was described previously^37^. Oocytes were injected with 50–70 nl of cRNA at a concentration of 1–3 ng/nl using a micro-injector (Drummond Scientific, Broomall, PA). The oocytes were incubated in a ND96 solution containing: NaCl, 96 mM; KCl, 2 mM; CaCl_2_, 1.8 mM; MgCl_2_, 2 mM and HEPES, 5 mM (pH 7.4), supplemented with 50 mg/l gentamycin sulfate and 0.5 mM theophylline. Oocytes were stored for 1–5 days at 16 °C until sufficient expression of Na_V_s was achieved.

### Electrophysiology

Whole-cell currents from oocytes were recorded at room temperature (18–22 °C) by the two-electrode voltage clamp technique using a GeneClamp 500 amplifier (Molecular Devices, Sunnyvale, CA) controlled by a pClamp data acquisition system (Molecular Devices). Oocytes were placed in a bath containing ND96 solution. Voltage and current electrodes were filled with 3 M KCl, and the resistances of both electrodes were kept between 0.7 and 1.5 MΩ). The elicited currents were sampled at 20 kHz and filtered at 2 kHz using a four-pole, low pass Bessel filter. To eliminate the effect of the voltage drop across the bath grounding electrode, the bath potential was actively controlled by a two-electrode bath clamp. Leak subtraction was performed using a-P/4 protocol. Whole-cell current traces were evoked every 5 s by a 100-ms depolarization to the voltage corresponding to the maximal activation of the Na_V_-subtype in control conditions, starting from a holding potential of −90 mV. Concentration-response curves were constructed by adding different toxin concentrations directly to the bath solution. The percentage of Na_V_ modulation was plotted against the logarithm of the applied concentrations and fitted with the Hill equation: *y* = 100/[1 + (*IC*_50_/[toxin])^*h*^], where *y* is the amplitude of the toxin-induced effect, *IC*_50_ is the toxin concentration at half maximal efficacy, [toxin] is the toxin concentration and *h* is the Hill coefficient. To investigate the effects on the voltage dependence of activation, current traces were induced by 100-ms depolarizations from a holding potential of −90 to 65 mV with 5-mV increments. To investigate the effects on the steady-state inactivation process, oocytes were depolarized using a standard two-step protocol. From a holding potential of −90 mV, 100-ms prepulses were generated, ranging from −90 to 65 mV with 5-mV increments, immediately followed by a 100-ms test pulse to 0 mV. Data were normalized to the maximal Na^+^ current amplitude, plotted against prepulse potential and fitted using the Boltzmann equation: *I*_Na_/*I*_max_ = [(1 - *C*)/(1 + exp((*V* - *V*_h_)/*k*))] + *C*, where *I*_max_ is the maximal *I*_Na_, *V*_h_ is the voltage corresponding to half-maximal inactivation, *V* is the test voltage, *k* is the slope factor, and *C* is a constant representing a non-inactivating persistent fraction (close to zero in control). Comparison of two sample means was made using a paired Student’s *t* test (p < 0.05). All data was analyzed using pClamp Clampfit 10.0 (Molecular Devices^®^, Downingtown, PA) and Origin 7.5 software (Originlab^®^, Northampton, MA) and is presented as mean ± standard error (S.E.M) of at least 3 independent experiments (n ≥ 3).

### Data availability

*Lineus longissimus* transcriptomic data is deposited in NCBI SRA: SRX1967959. Nemertide α−1 solution NMR structure is deposited in RCSB PDB: 6ENA.

## Electronic supplementary material

Supplemental Information
