# Supplementary material for: Peptide ion channel toxins from the bootlace worm, the longest animal on Earth
Source: Sci Rep. 2018 Mar 22;8:4596. doi: 10.1038/s41598-018-22305-w (PMC5864730; doi:10.1038/s41598-018-22305-w)
Supplement: Supplementary file 1 — Supplemental Information [file 41598_2018_22305_MOESM1_ESM.pdf]

SUPPLEMENTAL INFORMATION

to

Peptide ion channel toxins from the bootlace worm, the longest animal on earth

Erik Jacobsson[1], Håkan S. Andersson[2], Malin Strand[3], Steve Peigneur[4], Camilla Eriksson[1], Henrik Lodén[5], Mohammadreza Shariatgorji[5], Per E. Andrén[5], Eline K. M. Lebbe[4], K. Johan Rosengren[6], Jan Tytgat[4], Ulf Göransson[1]

[1]Division of Pharmacognosy, Department of Medicinal Chemistry, Biomedical Center, Uppsala University, Box 574, SE-751 23 Uppsala, Sweden
[2]Linnaeus University Centre for Biomaterials Chemistry, Department of Chemistry and Biomedical Sciences, Linnaeus University, Kalmar, Sweden
[3]Swedish Species Information Centre, Swedish University of Agricultural Sciences, Uppsala, Sweden
[4]Toxicology & Pharmacology, University of Leuven (KU Leuven), O&N 2, PO Box 992, Herestraat 49, 3000 Leuven, Belgium
[5]Biomolecular Mass Spectrometry Imaging (BMSI), National and SciLifeLab Resource for Mass Spectrometry Imaging, Department of Pharmaceutical Biosciences, Biomedical Center, Uppsala University, Box 591, SE-751 24 Uppsala, Sweden
[6]School of Biomedical Sciences, The University of Queensland, Brisbane, QLD 4072, Australia

SUPPLEMENTAL DATA ITEMS

Figure S1

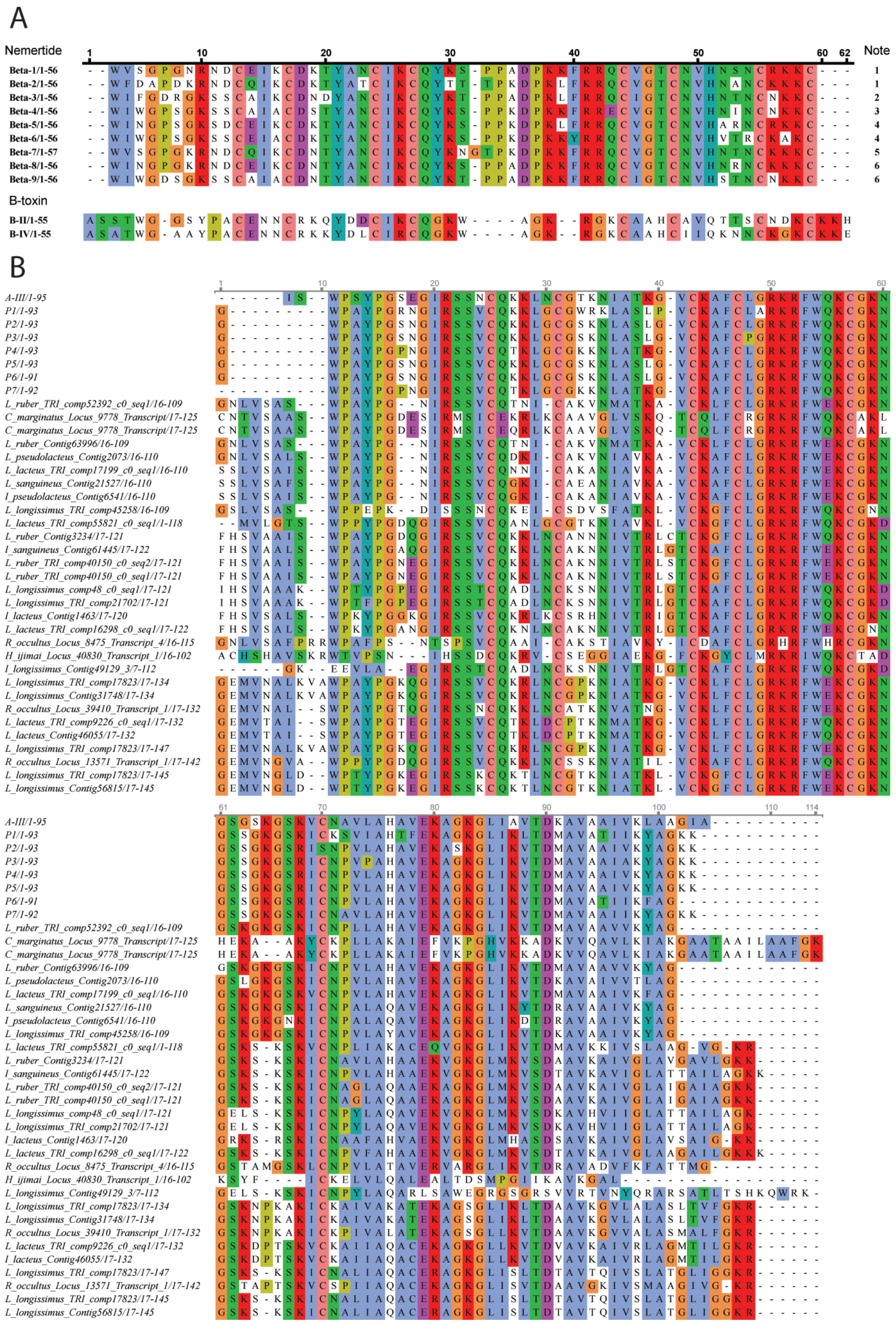

Figure S1. Peptide toxins identified by transcriptome mining. (**A**) β–nemertides. Clustal alignment of mature part as proposed by evidence of β-1 on peptide level. The B-toxins are displayed for reference. The clustal color scheme was used. Notes: the sequences were found in the following species: 1: *L. longissimus*, and *L. ruber*. 2: *L. longissimus* 3: *L. lacteus* 4: *L. pseudolacteus* 5: *L. ruber* 6: L. *sanguineus* (**B**) Parborlysins and cytolysin A-III analogs. Alignment of parborlysin 1-7, cytolysin A-III, and related sequences from the combined nemertean transcriptomes. The pairwise alignment identity ranges from 47.7 to 75.6% compared to parborlysin 1, and between 96.7 and 78.2% among the parborlysins themselves. The clustal color scheme was used in the alignment.

Figure S2.

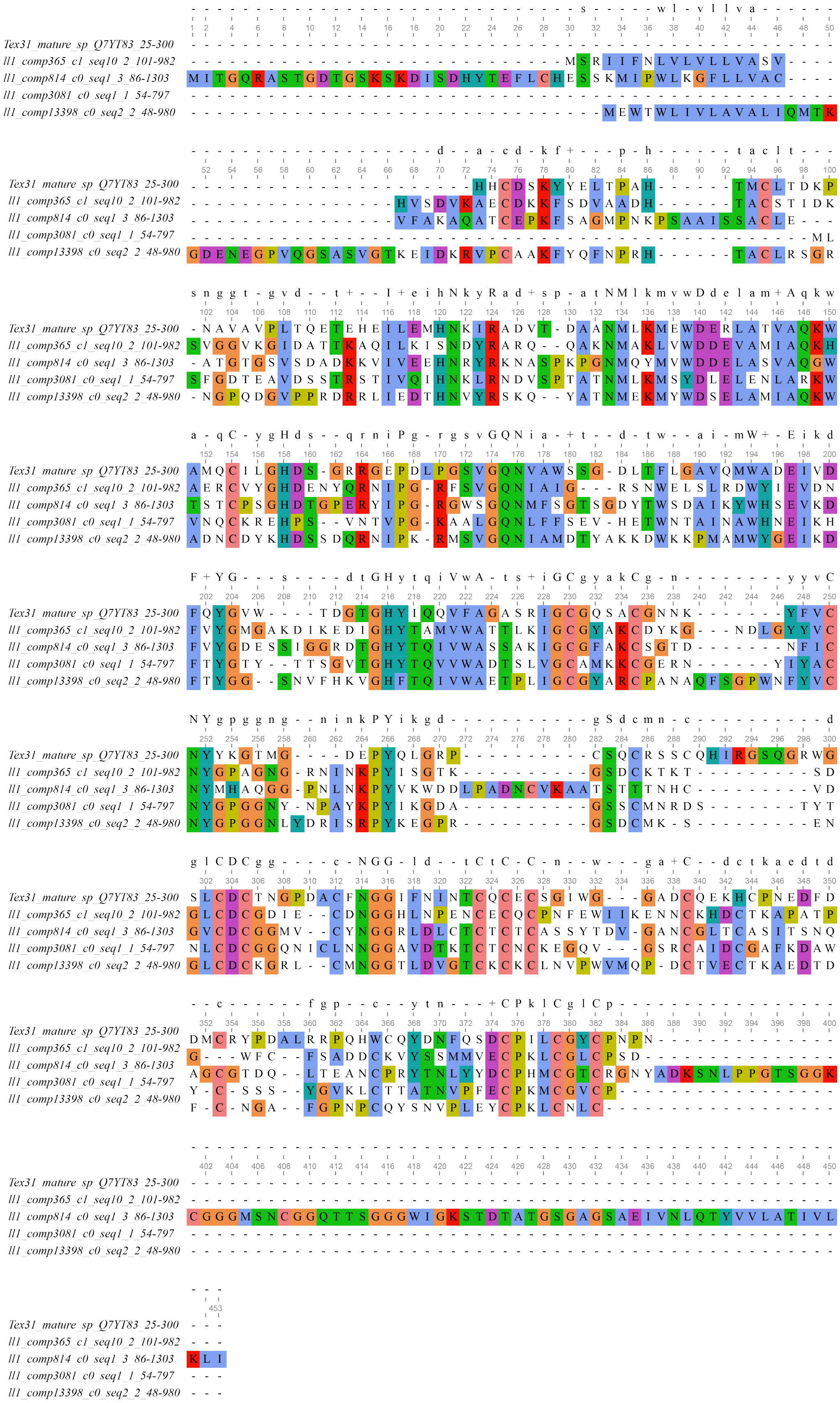

Figure S2. Sequences with similarity to the proposed peptide maturation enzyme Tex31 (top sequence for comparison) found in the *L. longissimus* transcriptome.

Figure S3

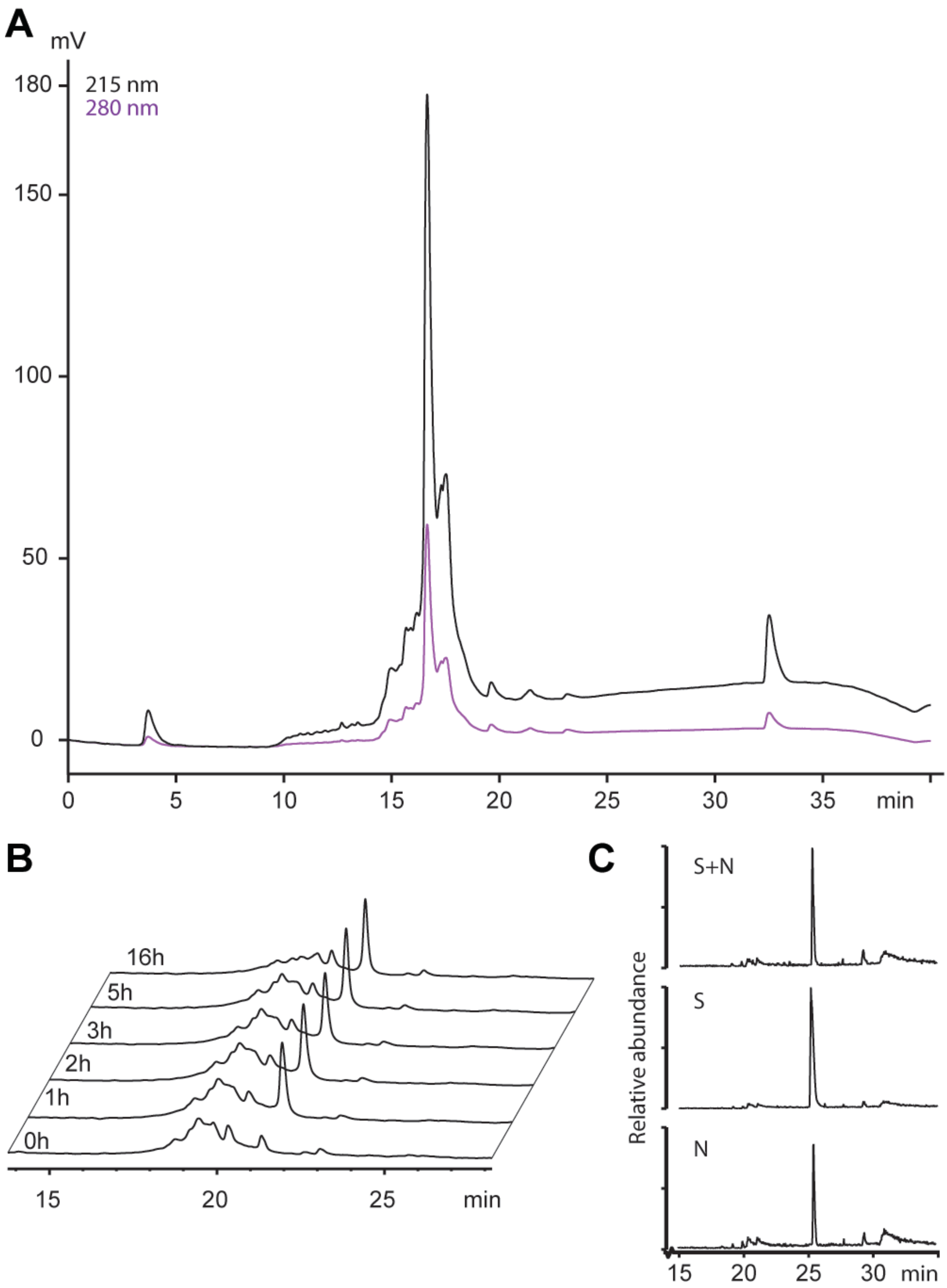

Figure S3. Purification, oxidative folding of α-1 and co-injection of native and synthetic peptide. ***(A)*** Analytical LC-UV trace of the crude cleaved α-1 peptide. Black: 215 nm, magenta: 280 nm. (**B**) RP-HPLC-UV of the folding process from 0 to 16 hours after folding initiation, all traces were recorded at 215 nm. (**C)** UPLC-MS analyses of folded synthetic and isolated native peptide demonstrating identical folds and purity of compounds. At the top, co-injection of synthetic α-1 (S) and native (N) and individual traces (separate injections) of S and N are shown below.

Figure S4

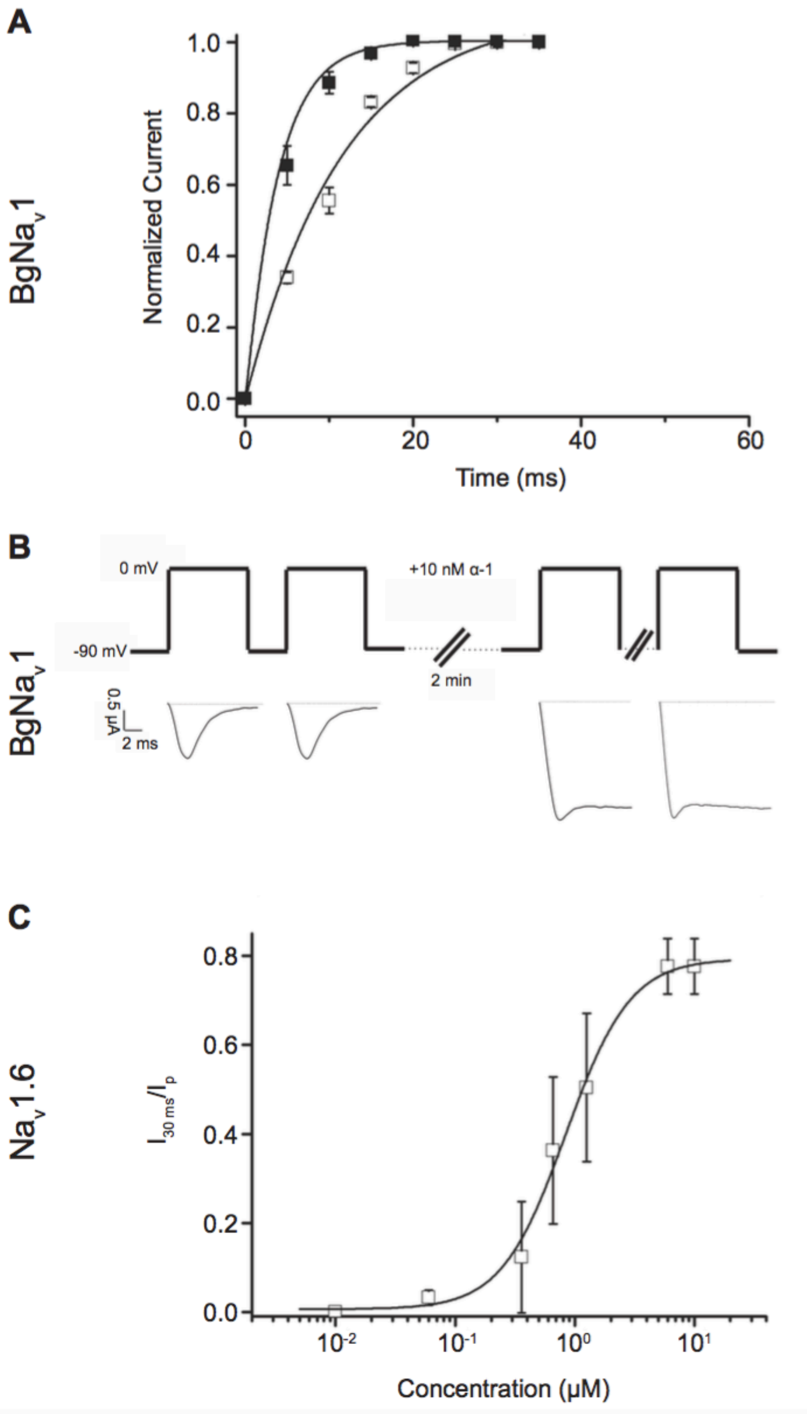

Figure S4. Recovery, state dependence and concentration-response of nemertide α-1 binding. (**A**) Recovery from inactivation of BgNa$_v$1 in control (open symbols) and in the presence of 10 nM α-1 (closed). Nemertide α-1 significantly enhanced the recovery from inactivation. τ values yielded 11.6 ± 0.4 ms and 3.8 ± 0.5 ms in control and after application of 10 nM α-1, respectively. (**B**) State dependence of inhibition. Nemertide α-1 does not require the open state of the BgNa$_v$1 channel to bind. **(C)** Concentration-response curve for Na$_V$1.6 indicating the concentration dependence of the α-1 induced effect. EC_{50} was determined to 0.8 ± 0.1 µM.

Table S1. Available nemertean transcriptomes and number of contigs (17 species).

Species	Class or Subclass/order	Number of contigs	Accession number(s)
Argonemertes australiensis	*Hoplonemertea/ Monostilifera*	43,999[1]	SRX646169
Baseodiscus unicolor	*Heteronemertea/-*	89,321[1]	SRX644738
Carinoma hamanako	*Palaeonemertea/-*	70,286[1]	SRX643224 (2 runs)
Cephalothrix hongkongiensis	*Palaeonemertea/-*	76,507[2]<br>73,445[1]<br>122,233[3]	SRX205320
Cephalothrix linearis	*Palaeonemertea/-*	67,317[3]	SRX534866–SRX534868
Cerebratulus marginatus	*Heteronemertea/-*	109,947[2]<br>117,305[1]<br>194,257[3]	SRX205323
Hubrechtella ijimai	*Palaeonemertea/-*	110,394[1]	SRX644663 (2 runs)
Lineus longissimus	***Heteronemertea/-***	79,452[4]<br>**81,597***	SRX565176–SRX565181<br>**SRX1967959***
Lineus ruber	*Heteronemertea/-*	156,014[4]	SRX565182–SRX565183
Ramphogordius lacteus	*Heteronemertea/-*	96,066[4]	SRX565174–SRX565175
Malacobdella grossa	*Hoplonemertea/ Monostilifera*	49,596[1]<br>109,120[3]	SRX646170[2]<br>SRX731465[3]

Nipponnemertes sp.	*Hoplonemertea/ Monostilifera*	28,772[1]	SRX647389
Paranemertes peregrina	*Hoplonemertea/ Monostilifera*	30,456[1]<br>99,203[3]	SRX646171<br>SRX731466
Protopelagonemertes beebei	*Hoplonemertea/ Polystilifera*	29,295[1]	SRX646186
Riseriellus occultus	*Heteronemertea/-*	91,728[1]	SRX644742
Tubulanus punctatus	*Palaeonemertea/-*	4,217[1]	SRX643211
Tubulanus polymorphus	*Palaeonemertea/-*	79,313[3]	SRX732127

Classification follows the World Register of Marine Species (http://www.marinespecies.org). *Current study.

Table S2. NMR spectroscopy statistics. Solution NMR spectroscopy statistics for the 20 selected models with lowest MolProbity value of the 50 models calculated with lowest overall energies.

**Energies (kcal/mol)**	
Overall	-1017.514 ± 15.234
Bonds	10.876 ± 0.941
Angles	34.851 ± 3.348
Improper	14.935 ± 2.003
van der Waals	-121.993 ± 4.921
NOE	0.065 ± 0.024
cDih	0.076 ± 0.106
Dihedral	130.507 ± 1.344
Electrostatic	-1086.831 ± 16.479

**MolProbity statistics**	
Clashes (0.4 Å/1000 atoms)	12.968 ± 3.825
Poor rotamers	2.502 ± 2.493
Ramachandran outliers	0.000 ± 0.000
Ramachandran favoured	99.465 ± 1.308
MolProbity score	1.911 ± 0.181
MolProbity score percentile	79.450 ± 8.525
Residues with bad bonds	0 ± 0
Residues with bad angles	0 ± 0

**Atomic r.m.s.d.c**	
Mean global backbone	0.39 ± 0.09 Å
Mean global heavy	1.03 ± 0.22 Å

**Distance restraints**	
Intraresidue (i – j = 0)	126
Sequential (|i – j| = 1)	109
Medium range (|i – j| <5)	47

Long range (|i – j| >)	105
Hydrogen bonds	24
Total	411

**Dihedral angle restraints**	
ϕ	20
Ψ	18
χ1	17
Total	55

**Violations from experimental restraints**	
Total NOE violations exceeding 0.2 Å	1 (0.202)
Total dihedral violations exceeding 3.0 °	0

MolProbity score is a value normalized to be in the scale of X-ray resolution and combines clashes, rotamers, and Ramachandran values; a lower value is better than a higher.

Table S3. Effect of nemertide a-1 injection in *B. dubia*.

Avg. dose (µg/kg)	Avg. dose nmole/kg	Avg. weight (g)	% Unaffected
control	control	1.24	80
0.52	0.16	1.03	80
1.04	0.31	1.26	80
2.18	0.66	1.50	100
7.13	2.15	1.13	100
*14.67	4.43	1.03	0
16.96	5.12	1.24	0
41.94	12.68	1.21	0
212.23	64.14	1.22	0
420.10	126.96	1.19	0

All concentrations were injected (10µl) in five (n=5) animals. Control: MilliQ water. *injection volume 7.5 µl (2µg/ml).

SUPPLEMENTAL EXPERIMENTAL PROCEDURES

Collection of *Lineus longissimus*. Living specimens of *L. longissimus* were collected and identified at the west coast of Sweden (Kosterfjord, 35 m depth) by Dr Malin Strand, Tjärnö Lovén Center, who also identified the specimens. Mucus was collected by placing specimens in a small container containing seawater and gently agitating the animal with a glass rod. Mucus was then collected and lyophilized. One specimen was cut into pieces, which were either flash-frozen in liquid nitrogen or placed in RNA-later® solution. The flash-frozen samples were stored at -80°C and the RNA-later® preserved samples were stored at -20°C, after overnight storage at 4°C, until further processing.

Peptide Isolation. The lyophilized mucus from one collection was dissolved in 12.5 ml 30% acetonitrile (AcN) in water and 0.1% formic acid (FA). Aliquots of 2.5 ml were desalted using size exclusion chromatography (SEC; PD-10, GE Healthcare). The high molecular weight eluate was collected and lyophilized before being redissolved in 10% AcN, 0.1% FA in water, and subjected to RP-HPLC on a Phenomenex Jupiter column (5µ C18 300Å, 250x4.6 mm) using a Shimadzu LC20 system equipped with a UV-detector. The gradient ranged from 5% AcN, 0.05% trifluoroacetic acid (TFA) to 55% AcN over 25 minutes. The three main peptides were subjected to quantitative amino acid analysis at the Amino Acid Analysis Center, Department of Biochemistry, Uppsala University.

LC-MS/MSMS and Peptide Sequencing. Peptides were reduced and alkylated using dithiothreitol (DTT) and iodoacetamide (IAM). Alkylated peptides were desalted using SEC, and digested with trypsin, chymotrypsin and endoproteinase Glu-C, in separate experiments, prior to MS-sequencing[5]. In short, dry, reduced and alkylated peptide was dissolved in 50 mM NH_4HCO_3 solution containing 4 µg/ml enzyme. The solution was incubated at 37 °C overnight prior to LC-MS and LS-MSMS analyses. Peptides were analysed using UPLC-QToF nanospray MS (Waters nanoAcquity, QToF Micro; 75 µm x 250 mm 1.7 µm BEH130 C18). The LC gradient ranged from 1% to 90% AcN (0.1% FA) over 50 minutes at a flow rate of 0.300 µl/min. Detection was done in positive ion mode, and data was collected between m/z 200-2000. The mass spectrometer was operated under MassLynx v. 4.1. Data directed analysis (DDA) was used for MSMS. The survey scan window was set to 200-2500 m/z and MSMS scan to 50-2000 m/z. The collision energy profiles ranged from 25-70 V.

After reduction and alkylation, peptide masses were increased with 348 Da for the two smaller compounds and 464 Da for the larger one. These increments in mass correspond to the presence of three and four disulfide bonds, respectively. In the MSMS analyses, some fragments of α-1 and α-2 showed identical masses and retention times, demonstrating homology between peptides. Combined, the m/z 463^{2+} and 679^{2+} fragments revealed identical 14-residue long sequences. Other fragments differed between peptides, including two ions with Δ 47.95: the tryptic 701^{2+} fragment of α-1 and 677^{2+} of α-2. MSMS sequencing of these fragments showed that these peptide fragments differ by a Phe to Val substitution (Δ 48.00), as shown in Figure 3. In total, MSMS sequencing revealed 20 out of 31 residues of α-1 and α-2. No sequence could be determined for β-1 by MSMS alone. The presence of the two Hyp-residues was unambiguously confirmed by comparing retention times and MS/MSMS spectra of reduced, and reduced and alkylated, peptides with and without modified Pro-residues.

Total RNA Extraction and Transcriptome Analyses. Total RNA was extracted from both flash-frozen and samples stored in RNAlater®, using Qiagen AllPrep DNA/RNA Mini Kit. The combined total RNA was sent to Macrogen (Korea) for Illumina HiSeq 2000 based RNA-seq paired end analysis, and assembled by Macrogen using Trinity (v 2011-11-26) [6]. The assembled transcriptome was either translated into protein sequences using the EMBOSS getorf tool as utilized in the graphic user interface eBioX (v. 1.5.1), or for preparation of local nucleotide NCBI BLAST+ databases through Unipro uGENE's (v. 1.14.0) [7] implementation of NCBI BLAST+. The sequenced tryptic/chymotryptic peptides were used as query in tBLASTn or BLASTp BLAST+ searches in the local *L. longissimus* transcriptome databases to confirm and complete the sequence. The α-1, α-2 and β-1 sequences were blasted against public generalistic databases (NCBI, UniProt) and the specialized databases Conoserver [8], and Arachnoserver [9]. The latter two databases are focused on toxin-like

peptides from *Conus* spp. and arachnoids respectively. The ConoPrec tool from conoserver was used to predict and classify the full precursor sequences according to Conoserver standards.

Publically available transcriptomic data (Table S1) from *Nemertea spp*. was downloaded from ftp://popphyl.univ-montp2.fr/contigs/ (accessed 2014-11-19): *Lineus lacteus*, *L. longissimus*, *L. pseudolacteus*, *L. ruber*, and *L. sanguineus*)[4]; http://figshare.com/articles/Nemertean_Trinotate_annotation_reports/1203580 (accessed 2015-10-14) *Cephalothrix hongkongiensis*, *C. linearis*, *Cerebratulus marginatus*, *Lineus lacteus*, *L. longissimus*, *L. ruber*, *Malacobdella grossa*, *Paranemertes peregrina*, and *Tubulanus polymorphus*)[3]. The assembled transcriptomes of *Argonemertes australiensis*, *Baseodiscus unicolor*, *Carinoma hamanako*, *Cephalotrix hongkongiensis*, *Cerebratulus marginatus, Hubrechtella ijimai*, *M. grossa*, *Nipponemertes sp*., *Riseriellus occultus*, *Paranemertes peregrina, Protopelagonemertes beebei*, and *T. punctatus*, were kindly provided by Andrade[1]. All sequences were combined in a single fasta file and the combined database was mined using BLAST+ and fuzz-pro/tran[1].

MALDI-MSI Analysis. The frozen *L. longissimus* tissues were cut using a cryostat-microtome (Leica CM3050S; Leica Microsystems, Welzlar, Germany) at a thickness of 14 µm, thaw-mounted onto conductive indium tin oxide (ITO) glass slides (Bruker Daltonics), and stored at −80°C. Sections were dried gently under a flow of nitrogen and desiccated at room temperature for 15 min, after which they were imaged optically using a photo scanner (Epson perfection V500). The samples were then coated with 2,5-dihydroxybenzoic acid (DHB) (35 mg/ml in 50% AcN, 0.2% TFA) using an automatic sprayer (TM-Sprayer; HTX Technologies, Carrboro, NC). MALDI-MSI experiments were performed using a MALDI-TOF/TOF (Ultraflextreme, Bruker Daltonics, Bremen Germany) mass spectrometer with a Smartbeam II 2 kHz laser operated in positive ion mode. The laser power was optimized before the start of the imaging experiment and then held constant during the MALDI-MSI analysis. Purified peptides α-1, α-2 and β-1 were spotted on one of the sections as an *in-situ* reference to establish the masses for the peptides in this system setup.

Peptide Synthesis. Nemertide α-1 was assembled on a TentaGel XV HMPA resin (0.21 mmol/g, 0.05 mmol scale) using Fmoc-based solid-phase peptide synthesis (SPPS). The C-terminal residues HypHypAsnGln were coupled manually. 10 equivalents (eq.) of Gln were coupled using 5 eq. DIC and 0.1 eq. DMAP. 6 eq. Asn and 2 eq. Hyp were coupled using HBTU and DIPEA (6, 9 eq. and 2, 3 eq. respectively). 2 eq. Fmoc-Leu-Ser($\psi^{Me,Me}$Pro)-OH were coupled in position 11-12 (LeuSer) to prevent peptide chain aggregation (2 eq. HBTU and 3 eq. DIPEA). Remaining residues were assembled using automated microwave-assisted SPPS. Due to the high swelling nature of the resin, 4 times the standard (5eq. amino acid, 5 eq. HBTU, 10 eq. DIPEA) scale was used to ensure full reagent coverage of the resin. For Fmoc-deprotection 20% (v/v) piperidine in DMF was used. The synthesis procedure was repeated to yield 0.1 (theoretical) mmol of resin-bound α-1.

Simultaneous cleavage from resin and side-chain protection groups was performed by stirring the resin in TFA/TIPS/ H_2O (95:2.5:2.5 v/v) for 2 hours. The TFA was evaporated using N_2 and the peptide was precipitated with cold diethyl ether. The peptide was collected by partitioning with 50% AcN/0.1% TFA followed by lyophilization. An analytical HPLC trace of the crude peptide is shown in Fig. S3A. Crude peptide (yield: 92%) was subjected to oxidative folding in a GSH:GSSG 2 mM: 0.4 mM in 0.1 M NH_4HCO_3 (pH 8.5), containing 20% (v/v) isopropanol. The folding mixture was diluted to a final concentration of 6% isopropanol and the peptide was purified (final yield: 9%) using RP-HPLC-UV with a Phenomenex Jupiter C18 column (250x10 mm, 5µ) and a gradient from 5% AcN, 0.05% TFA to 97% AcN, 0.05% TFA in 45 minutes, at a flow rate of 4 ml/min. An aliquot of folded and purified peptide was co-injected with native α-1 into LC-MS to prove the conformity between the synthetic and native peptide, Fig. S3C.

NMR Structure Determination and Structural Comparison. For NMR analysis synthesized α-1 was dissolved in 10% D_2O in H_2O, and data collected on a Bruker Avance 600 MHz spectrometer equipped with a cryoprobe. 2,2-Dimethyl-2-silapentane-5-sulfonate (DSS) was added and used as

internal standard (0.0 ppm). Two-dimensional spectra (*i.e.* TOCSY, NOESY, ^{13}CHSQC, ^{15}NHSQC) were recorded at 298 K. TOCSY spectra were collected at five temperatures 288-308 with 5 K increments Structural alignment of the solution NMR structure of α-1 against the Protein Data Bank through DALI server[10] identified the closest match with respect to Z score, as the human liver expressed antimicrobial peptide-2, LEAP-2 (Z: 3.7, (rmsd: 1.1, 21% sequence identity) followed by two ICK spider toxins, ω-HXTX-Hv1a (previously; ω-ACTX- Hv1a) Z: 3.4 (rmsd: 0.8, 30% sequence identity) and κ-TRTX-Scg1a (previously; SGTx1) Z: 3.3 (rmsd: 1.6, 20% sequence identity).

***Blaptica dubia* Assay**

50 juvenile specimens of *Blaptica dubia* cockroaches were acquired at a local store (Herper's choise (sic), Uppsala), and toxicity was assessed following the strategy outlined by Liu *et al* [11]. Single doses consisting of 10 µl peptide solution was injected between the 4th and 5th sternite, using a pointed GC Hamilton syringe (25 microliter model nr. 702). The cockroaches were then subdivided into groups of five individuals per concentration and housed in ventilated plastic containers containing hibiscus leaves for 24h post injection. Cockroaches that were able flip over from an upside-down placement were considered unaffected. After the experiment all cockroaches were weighted, and average doses were calculated (0, 0.5, 1.0, 2.2, 7.1, 14.7, 17.0, 41.9, 212.9, 420.1 µg/kg). All solutions were prepared in MilliQ water, including the control.

SUPPLEMENTAL REFERENCES

1 Andrade, S. C. *et al.* A transcriptomic approach to ribbon worm systematics (nemertea): resolving the pilidiophora problem. *Molecular biology and evolution* **31**, 3206-3215, doi:10.1093/molbev/msu253 (2014).

2 Riesgo, A. *et al.* Comparative description of ten transcriptomes of newly sequenced invertebrates and efficiency estimation of genomic sampling in non-model taxa. *Frontiers in zoology* **9**, 33, doi:10.1186/1742-9994-9-33 (2012).

3 Whelan, N. V., Kocot, K. M., Santos, S. R. & Halanych, K. M. Nemertean toxin genes revealed through transcriptome sequencing. *Genome Biol Evol* **6**, 3314-3325, doi:10.1093/gbe/evu258 (2014).

4 Romiguier, J. *et al.* Comparative population genomics in animals uncovers the determinants of genetic diversity. *Nature* **515**, 261-263, doi:10.1038/nature13685 (2014).

5 Carstens, B. B. *et al.* Isolation, Characterization, and Synthesis of the Barrettides: Disulfide-Containing Peptides from the Marine Sponge Geodia barretti. *J Nat Prod* **78**, 1886-1893, doi:10.1021/acs.jnatprod.5b00210 (2015).

6 Grabherr, M. G. *et al.* Full-length transcriptome assembly from RNA-Seq data without a reference genome. *Nature biotechnology* **29**, 644-652, doi:10.1038/nbt.1883 (2011).

7 Okonechnikov, K., Golosova, O., Fursov, M. & team, U. Unipro UGENE: a unified bioinformatics toolkit. *Bioinformatics* **28**, 1166-1167, doi:10.1093/bioinformatics/bts091 (2012).

8 Kaas, Q., Yu, R., Jin, A. H., Dutertre, S. & Craik, D. J. ConoServer: updated content, knowledge, and discovery tools in the conopeptide database. *Nucleic acids research* **40**, D325-330, doi:10.1093/nar/gkr886 (2012).

9 Herzig, V. *et al.* ArachnoServer 2.0, an updated online resource for spider toxin sequences and structures. *Nucleic acids research* **39**, D653-657, doi:10.1093/nar/gkq1058 (2011).

10 Holm, L. & Rosenstrom, P. Dali server: conservation mapping in 3D. *Nucleic acids research* **38**, W545-549, doi:10.1093/nar/gkq366 (2010).

11 Liu, K. *et al.* Venom from the spider Araneus ventricosus is lethal to insects but inactive in vertebrates. *Toxicon* **115**, 63-69 (2016).
